# The NEL Family of Bacterial E3 Ubiquitin Ligases

**DOI:** 10.3390/ijms23147725

**Published:** 2022-07-13

**Authors:** Andrea Bullones-Bolaños, Joaquín Bernal-Bayard, Francisco Ramos-Morales

**Affiliations:** Departamento de Genética, Facultad de Biología, Universidad de Sevilla, 41012 Sevilla, Spain; abullones@us.es (A.B.-B.); jbbayard@us.es (J.B.-B.)

**Keywords:** E3 ubiquitin ligases, type III secretion, effectors, *Shigella*, *Salmonella*, *Sinorhizobium*, *Ralstonia*

## Abstract

Some pathogenic or symbiotic Gram-negative bacteria can manipulate the ubiquitination system of the eukaryotic host cell using a variety of strategies. Members of the genera *Salmonella*, *Shigella*, *Sinorhizobium*, and *Ralstonia*, among others, express E3 ubiquitin ligases that belong to the NEL family. These bacteria use type III secretion systems to translocate these proteins into host cells, where they will find their targets. In this review, we first introduce type III secretion systems and the ubiquitination process and consider the various ways bacteria use to alter the ubiquitin ligation machinery. We then focus on the members of the NEL family, their expression, translocation, and subcellular localization in the host cell, and we review what is known about the structure of these proteins, their function in virulence or symbiosis, and their specific targets.

## 1. Introduction

### 1.1. Type III Secretion Systems

Many Gram-negative bacteria that are pathogens or symbionts of plants or animals use type III secretion systems (T3SSs) to inject proteins known as effectors into the cytosol of eukaryotic host cells [1,2]. These systems are molecular syringes, evolutionarily related to flagella, that use a single-step secretion mechanism to cross the inner and outer bacterial membranes and the host cell membrane [3].

There are more than 500 experimentally validated T3S effectors, and over 8000 were identified as candidate effectors by sequence similarity search [4]. Effectors are extremely diverse, although they share some common structural features [5]. They contribute to the manipulation of the host using a variety of eukaryotic targets located in different cellular compartments [6]. These proteins exhibit a modular structure with several domains or motifs [7]. The secretion domain is usually located in the first 25 N-terminal residues, and some effectors have a chaperone binding domain between residues 50 and 150 [8], although specific secretion through the injectisome may also depend in some cases on the C-terminal region [9,10,11,12]. Functional domains are typically localized to the central and C-terminal portions, and many effectors are chimeras of other known effectors, or an effector and another protein [13]. Effectors may contain domains or motifs involved in subcellular targeting, including mitochondrial targeting sequences, membrane-targeting motifs, and nuclear localization sequences, and in protein–protein interaction. Among the host processes that are manipulated by the activity of effector domains are caspase activation, actin nucleation, microtubule polymerization, regulation of G proteins, AMPylation, ADP-ribosylation, cAMP synthesis, proteolysis, lipid degradation, phosphorylation, and ubiquitination [7]. This review focuses on a family of bacterial E3 ubiquitin ligase effectors whose secretion and activity have been studied in *Salmonella*, *Shigella*, *Sinorhizobium* (*Ensifer*), and *Ralstonia*.

**Figure 1:**
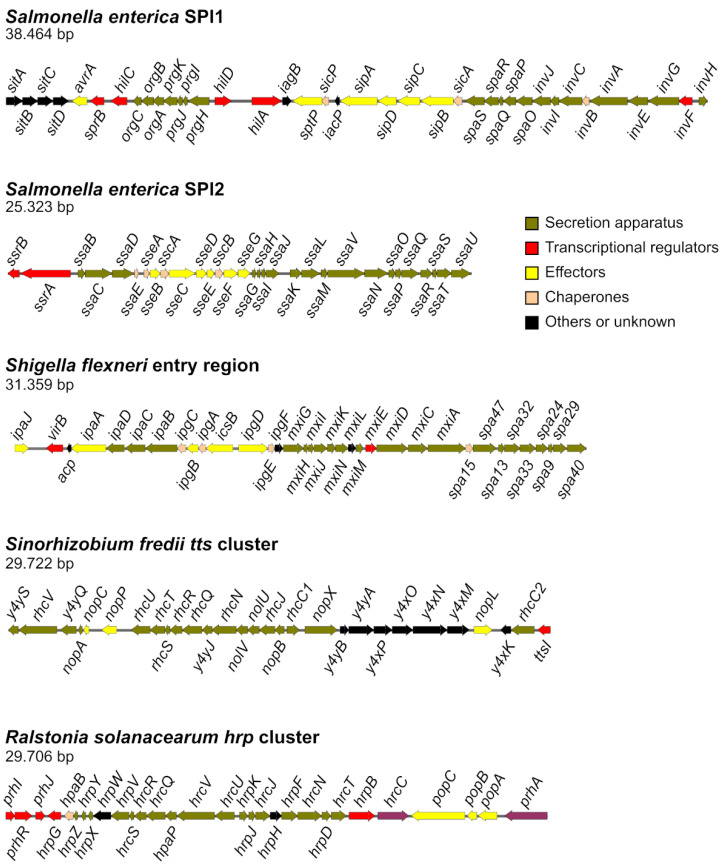
Clusters of genes that encode T3SSs. SPI1 and SPI2 from *S. enterica* serovar Typhimurium, the entry region from *S. flexneri*, the *tts* cluster from *S. fredii*, and the *hrp* cluster from *R. solanacearum*.

*Salmonella enterica* is a species of facultative intracellular bacterial pathogens that belongs to the family *Enterobacteriaceae* in the class Gammaproteobacteria. These bacteria can infect humans and other animals, causing diseases ranging from gastroenteritis to systemic infection, depending on the serovar–host combination [14]. *S. enterica* possesses two distinct T3SS, T3SS1 and T3SS2, whose structural components and some of their effectors are encoded by two *Salmonella* pathogenicity island: SPI1 and SPI2, respectively (Figure 1). T3SS1 acts at the beginning of infection and promotes the invasion of epithelial cells in the intestine and many other host cell types in vitro [15]. T3SS2 is expressed inside the *Salmonella* containing vacuole (SCV), the typical intracellular niche of these bacteria, and is necessary for intracellular survival and systemic dissemination [16]. Together, the two T3SS of *S. enterica* secrete over 30 effectors [17].

*Shigella flexneri* is one of four species of human restricted pathogens classified in the genus *Shigella*, another member of the family *Enterobacteriaceae*. Shigellosis, or bacillary dysentery, is a gastrointestinal infection with clinical symptoms that range from mild watery diarrhea to bloody mucoid diarrhea with abdominal cramps and fever [18]. *Shigella* enters epithelial cells, but, unlike *Salmonella*, escapes the vacuole in which it is internalized, proliferates in the host cytoplasm, and induces actin polymerization that allows the movement of the bacterium and the dissemination to adjacent cells [19]. The T3SS apparatus and most of its effectors are encoded in a 230 kb virulence plasmid [20]. Genes encoding the structural components (*mxi-spa* genes) and some effectors and regulators of the system are located in a 31 kb pathogenicity island termed the entry region [20,21] (Figure 1). *Shigella* secretes about 30 effectors through this system, some involved in host cell invasion and some involved in dampening the host innate responses [22].

*Sinorhizobium fredii* is an Alphaproteobacteria of the *Rhizobiaceae* family [23]. These bacteria are capable of establishing nitrogen-fixing symbioses with more than 100 genera of legumes [24]. The three most studied strains of this species are NGR234, USDA257, and HH103. These strains differ in their nodulation host range and genome size and organization [25]. A functional T3SS gene cluster (*tts* cluster), T3SS-I, is located in the symbiotic plasmid of each strain, pNGR234a, pUSDA257, and psfHH103d, respectively [25,26,27] (Figure 1). Some of the proteins secreted through this system, known as nodulation outer proteins (Nops), have been characterized and are involved in the determination of host range and symbiotic efficiency [28].

*Ralstonia solanacearum* species complex belongs to class Betaproteobacteria and the family *Burkholderiaceae*. This is an economically important plant pathogenic bacterial species with a global distribution, wide host range, and long-lasting persistence in the soil that is responsible for bacterial wilt in more than 250 plant species [29]. The genome of *R. solanacearum* is organized in two replicons usually known as the chromosome and the megaplasmid [30]. The main determinant of virulence of these bacteria is the T3SS, encoded by the *hrp* (hypersensitive response and pathogenicity) cluster, a 23 kb region located in the megaplasmid [31] (Figure 1). This system allows translocation of effectors, known as *Ralstonia*-injected proteins (Rips), that are capable of subverting the defenses and modifying the metabolism of the host [32]. The T3SS is also essential for the elicitation of the hypersensitive response on non-host plants. This is a local defense reaction that induces necrosis of the infected tissue and prevents the multiplication of bacteria [33]. A genomic study on 140 strains identified more than 100 effectors, but only 16 were present in at least 95% of sequenced strains and were considered core effectors [34].

### 1.2. Ubiquitination Systems

Ubiquitin is a protein of 76 amino acids [35] that belongs to the superfamily of ubiquitin-like (Ubl) proteins. Members of this superfamily share structural similarities, although they do not have a high level of sequence identity [36]. Although Ubl proteins are widely distributed among all organisms, prokaryotes, and eukaryotes, ubiquitin is especially conserved in eukaryotes both structurally and functionally. At the C-terminal end, it has two glycine residues, which constitute an essential motif for its activity. At a functional level, the covalent binding of ubiquitin to target proteins during the process known as ubiquitination constitutes one of the most important cell signaling mechanisms to maintain cell homeostasis [37,38].

Ubiquitination is a reversible post-translational modification [39,40,41]. Currently, in addition to the well-described role for proteasome-mediated protein degradation, there are multiple cellular processes and signaling pathways, not related to proteolysis, where ubiquitination participates as a regulatory mechanism [42]. Ubiquitination occurs on lysine (K) residues; a substrate protein can be modified in a single lysine or in multiple lysines, which is known as monoubiquitination or multiubiquitination, respectively. Furthermore, ubiquitin has seven lysine residues (K6, K11, K27, K29, K33, K48 and K63) that, together with methionine 1 (M1), are susceptible to ubiquitination. This leads to the formation of ubiquitin polymers that can be very diverse depending on how ubiquitins are linked, and the modification of a substrate protein with ubiquitin polymers is known as polyubiquitination [43]. The diversity of physiological implications that ubiquitination has is partially explained by the variety of protein modifications in which ubiquitin may be involved. Furthermore, the type of link that ubiquitin establishes with the substrate can alter both protein activity and its location [44]. In fact, modifications such as K48-linked chains [45], K11-linked chains [46], and some monoubiquitinations [47] result in proteasome-mediated protein degradation. Contrarily, the addition of K63-linked chains does not have proteolytic consequences, but this modification is crucial for some signaling pathways and regulates processes as diverse as DNA repair [48], inflammation, and immune response, among others [49].

The linking of ubiquitin to a substrate requires the sequential participation of three enzymes that are essential components of the ubiquitination system [39]. The first step of the process consists of ubiquitin activation; this is carried out by the ubiquitin-activating enzyme (E1), and the reaction occurs in two steps and requires ATP. E1 activity leads to the formation of a thiol ester link between G76 from ubiquitin and cysteine from the active E1 site [50,51]. Subsequently, the E1-ubiquitin complex interacts with the ubiquitin-conjugating enzyme (E2), which promotes the transfer of ubiquitin from one enzyme to the other due to conformational changes that favor the approach of both active sites [37]. The final step of the process involves the interaction of the E2-ubiquitin complex with the ubiquitin ligase (E3), which carries out the transfer of ubiquitin to the substrate [37]. The E3s are responsible for selecting the final substrate for the ubiquitination reaction and catalyzing the formation of a peptide bond between the C-terminal G76 of ubiquitin and the amino group of the substrate lysine [37].

Classically, three families of E3 ubiquitin ligases are recognized: Really Interesting New Gene (RING), Homologous to E6AP C-Terminus (HECT), and RING Between RING (RBR). This classification is based on the specific structural characteristics and the ubiquitin transfer mechanism of the enzyme [37]. The RING E3 ligases promote the transfer of ubiquitin directly from E2 to the substrate, while the HECT and RBR ligases have a conserved cysteine residue where the ubiquitin binds before being transferred to the substrate, thus forming an intermediate E3-Ub. However, RBR ligases have interaction domains similar to those present in RING ligases [52,53].

Although the ubiquitination system is typically eukaryotic, some pathogenic bacteria have developed tools to manipulate, mimic, or hijack this system and enhance their virulence. Some bacteria encode effector proteins with ubiquitin ligase activity; some of these effectors belong to eukaryotic families of E3 ligases [54,55]. However, some others have structural and functional characteristics that are different from the existing families, which is why they have been grouped into a new family of E3 ligases called Novel E3 Ligase (NEL) [56,57]. At the structural level, most proteins belonging to the NEL family have two domains. In the N-terminal region, there is the leucine rich repeat (LRR) domain, which participates in the recognition and interaction with the substrate, while the C-terminal region harbors the NEL domain, which is the catalytic domain, since it contains a cysteine residue that allows the formation of an intermediate E3-ubiquitin, and is also responsible for the recognition of the E2-ubiquitin complex [55,58]. Both domains are connected through a flexible linker, which facilitates their spatial arrangement. Structural studies suggest that the LRR domain is involved in effector autoinhibition because, in the absence of substrate, it blocks the transfer of ubiquitin. The interaction of the LRR domain with the substrate promotes conformational changes that expose the active site and promote ubiquitin transfer [59].

As mentioned above, the typical ubiquitination system involves the sequential participation of enzymes E1, E2, and E3 to carry out the covalent binding of ubiquitin to a substrate protein. Although this mechanism is widely used in eukaryotes, it is not the only mechanism by which a protein can be ubiquitinated. In fact, some pathogenic bacteria have developed alternative ubiquitination systems independent of enzymes E1 and E2 [60]. Although this kind of effector is not very widespread, its existence evidences the biological and functional diversity within the ubiquitination process and highlights the importance of this process in cell regulation, as it is one of the routes targeted by bacterial pathogens for their own benefit.

## 2. Ubiquitination in Bacterial Pathogenesis

Host ubiquitin signaling plays a crucial role in cell defense mechanisms against incoming pathogens, mainly through a fine modulation of innate and adaptative immune responses, including a set of defensive strategies such as selective autophagy and phagosomal maturation, controlled activation of inflammatory signaling, and apoptosis [61,62]. Remarkably, although bacteria lack ubiquitin, these microorganisms have evolved strategies to hijack host ubiquitination machinery to destroy or inactivate key targets and evade the cell defense system [63]. For this purpose, bacterial pathogens utilize sophisticated secretion systems (T3SS or T4SS) to translocate protein effectors. Some effectors target and manipulate the host ubiquitination system, while other structurally mimic host E3 ligases or deubiquitinases (DUBs). In this section, we briefly show a few representative examples of manipulation of the mammalian ubiquitin machinery by bacteria, whereas in the next section we focus in depth on the NEL family of E3 ligases.

### 2.1. Ubiquitination and Bacterial Invasion and Adhesion

There are many examples of the role that the regulation of ubiquitination has on cytoskeleton rearrangement necessary for bacterial invasion of non-phagocytic cells via either the zipper or the trigger mechanisms. For example, host DUB UCH-L1 promotes the invasion of epithelial cells by *Listeria monocytogenes* and *S. enterica* by modifying the dynamics of the actin cytoskeleton [64]. Additionally, CDC42, a GTPase of the Rho family, is ubiquitinated during the invasion of epithelial cells by *Salmonella*, and this may represent a mode of regulation of GTPase activity in the course of infection [65]. An example of the role of ubiquitination in adhesion to host cells is found in enteropathogenic *E. coli* (EPEC), the causative agent of severe diarrhea in humans. This extracellular pathogen is able to colonize the intestinal mucosa, producing, attaching, and effacing lesions with the formation of actin-rich pedestals where bacteria adhere. Pedestal formation requires T3SS effectors such as Tir. Interestingly, EPEC encodes the HECT-type E3 ligase effector NleL, and this effector plays a role in down-modulating the pedestal formation. A more recent report showed that the primary substrate of NleL is c-Jun NH2-terminal kinase (JNK). Monoubiquitination at the Lys68 residue of JNK impairs its interaction with an upstream kinase, disrupting JNK activation [66].

### 2.2. Manipulation of the Innate Immune Response (NF-KB)

The innate immune system senses invading bacteria by detection of conserved bacterial components or pathogen-associated molecular patterns (PAMPs) by a set of pattern-recognition receptors (PRRs), including NOD-like receptors (NLR), Toll-like receptor (TLR), tumor necrosis factor receptor (TNFR) and interleukin-1 receptor (IL-1R) [67]. Several E3 ligases participate in the inflammatory signaling cascade initiated upon PRR activation. They belong to the family of TNFR-associated factors (TRAF) and contain an N-terminal domain with E3 ubiquitin ligase activity. They function as linkers between PRRs and the downstream signaling cascade that results in activation of NF-κB signaling, which in turns induces the expression of inflammatory cytokines or chemokines, such as IL-8, IL-1β, and TNF [68]. NF-κB activation is regulated by the IKK-complex, which is composed of IKKα, IKKβ, NEMO/IKKγ, and NF-κB inhibitor IκB. PRR activation by PAMPs provokes NEMO K63 polyubiquitination, leading to activation of the IKK complex, which phosphorylates IκB, leading to its polyubiquitination and posterior degradation by the proteasome [69]. NF-κB is then released and moves to the nucleus, where it induces the expression of inflammatory cytokines. Several animal pathogens, including *S. enterica* and *S. flexneri*, have evolved ubiquitin-related enzymes to downregulate the NF-κB-dependent inflammatory response.

*S. enterica* serovar Typhimurium has developed an arsenal of ubiquitin-related effectors including several NEL effectors that will be discussed in detail in the next section, one HECT-like E3 ligase (SopA), as well as two DUBs (SseL and AvrA). SopA ubiquitinates TRIM56 and TRIM65, two host RING E3 ubiquitin ligases that are essential for type I interferon signaling, and, therefore, modulates the inflammatory response [70]. AvrA inhibits host inflammatory responses by deubiquitination of IκBα and β-catenin, two inhibitors of the NF-κB pathway [71]. SseL also suppresses IκBα ubiquitination and degradation, preventing subsequent NF-κB activation [72]. In addition, SseL deubiquitinates ribosomal protein S3 (RSP3), a component of the eukaryotic 40S small ribosomal unit that also has a role as a component in NF-κB complexes guiding the NF-κB p65 subunit to specific gene promoters. This deubiquitination was restricted to K63 linkages and inhibited nuclear translocation of RSP3 [73]. Other authors, however, were unable to detect an involvement of SseL in the inhibition of host inflammatory responses [74].

*S. flexneri* secretes several NEL E3 ligase effectors that target and downregulate NF-κB signaling via their E3 ligase activity at different levels (see the next section). This pathogen also uses the T3SS effectors OspG and OspI to interfere with the host ubiquitination system. The kinase effector OspG binds to several E2 ubiquitin-conjugating enzymes, including UbcH5b and UbcH7, when these E2s are conjugated to ubiquitin, and prevents the proteasome-dependent degradation of IκBα [75]. The effector OspI deamidates the E2 enzyme Ubc13, leading to the disruption of TRAF6-catalyzed polyubiquitination and suppression of the pro-inflammatory diacylglycerol-CBM-TRAF6-NF-κB signaling pathway [76].

### 2.3. Manipulation of Defense-Associated Ubiquitination Machinery of Host Plants

Plant immune defense includes PAMP-triggered immunity (PTI) [77]. PTI responses involve the production of ROS, the activation of defense genes, and defense hormones. Effector-triggered immunity (ETI) is a second line of defense activated after detection of intrusive effectors through intracellular NLRs (nucleotide-binding domain leucine-rich repeat containing receptors or NOD-like receptors) [78]. ETI is often accompanied by the hypersensitive response mentioned above, a local cell death at the pathogen entry site [79]. Like animal pathogens, plant-interacting bacteria, both pathogenic and symbiotic, modulate the ubiquitination system for their benefit [80]. Some examples are given here, other than the NEL ligases that will be discussed in the next section.

*Pseudomonas syringae* is a well-studied phytopathogen that encodes the T3SS effector AvrPtoB, a RING/U-box E3 ligase involved in the inhibition of the plant immune response [81,82]. AvrPtoB targets the ubiquitin-26S proteasome system. It drives the ubiquitination and degradation of several PRRs (FLS2, BAK, CERK, EFR) and immune proteins (Pto, Fen, Prf) [80]. *Xanthomonas oryzae*, the causative agent of rice bacterial leaf blight, encodes an effector with E3 ligase activity, XopK, which is required for full pathogen virulence, and mediates the degradation of OsSERK2, a key regulator of rice immunity [83]. Finally, an interesting example of molecular mimicry used by some bacteria to subvert a host cellular process is pathogen-encoded F-box proteins. F-box are components, together with Skp1 and cullin1, of the SCF ubiquitin ligase complex that mediate polyubiquitination of target proteins and the subsequent proteasome-dependent protein degradation in eukaryotic cells [84]. An F-box-coding gene was found in *Agrobacterium tumefaciens*, a bacterial pathogen that causes crown gall disease in plants [85]. *Agrobacterium* translocates the coded effector, VirF, into plant cells [86] and hijacks the host SCF complex to facilitate bacterial infection. Many other viral and bacterial pathogens encode F-box-like effector proteins, including the plant pathogens *P. syringae, Pseudomonas savastanoi,* several species of the genus *Xanthomonas,* and *R. solanacearum* [87].

### 2.4. Subversion of Xenophagy

Autophagy is a fundamental cellular process that enables cells to engulf and digest portions of their cytoplasm in a regulated manner [88]. Xenophagy refers to the use of selective autophagy to eliminate intracellular pathogens. This process is an important ubiquitin-dependent innate immune mechanism [89]. Cytosolic bacteria are ubiquitin-coated, and this coating serves as a signal for selective autophagy leading to bacterial clearance. *S. enterica* is a well-studied model of intracellular bacteria targeted by xenophagy and of the strategies that bacteria can use to escape this host defense mechanism. Cytosolic *Salmonella,* as well as bacteria in SCVs damaged by the T3SS1, are polyubiquitinated with M1- and K63-linked ubiquitin chains that are recognized by autophagy receptors, leading to bacterial degradation by the autophagosome [65,90,91]. *Salmonella* and other intracellular pathogens have developed several mechanisms to subvert these host defense mechanisms. For example, the above-mentioned *S. enterica* serovar Typhimurium DUB SseL inhibits selective autophagy by eliminating ubiquitin aggregates of cytosolic *Salmonella* and SCV [92]. The T3SS1 effector AvrA from *S. enterica* serovar Enteritidis contributes to the inhibition of autophagy by decreasing expression of Beclin-1, a key regulator of autophagy. This effect is mediated through the inhibition of the JNK signaling pathway, but the authors also suggest an effect of AvrA on the ubiquitination of Beclin-1 [93]. Other *Salmonella* virulence factors interfering with autophagy are the T3SS2 effectors SpvB and SpvC and the T3SS1 effector SopF [94,95,96]. Phytopathogens also use multiple mechanisms to manipulate plant autophagy [97]. For instance, *Xanthomonas campestris* suppresses host autophagy by utilizing the T3SS effector XopL. XopL is an E3 ligase that ubiquitinates the autophagy component SH3P2 and mediates its proteasomal degradation. In addition, XopL is ubiquitinated in plants and degraded by NBR1-mediated selective autophagy, suggesting an early defense mechanism of the immune system [98].

## 3. E3 Ubiquitin Ligases of the NEL Family

### 3.1. Members of the Family

Typically, T3SS effectors belonging to this family share a domain structure composed of a central leucine-rich repeat (LRR) domain (InterPro domain IPR032674) and a C-terminal novel E3 ubiquitin ligase (NEL) domain (IPR029487) (separated from the LRR domain by a linker or intervening region), in addition to the N-terminal secretion motif. InterPro identifies more than 1000 proteins with this architecture and more than 6000 proteins that possess the NEL domain alone based on sequence similarity [99]. However, only a few of all these proteins have been experimentally shown to be T3SS effectors (Table 1). NEL effectors with LRR domains have been studied in the genera *Salmonella*, *Shigella*, and *Sinorhizobium*, whereas members of the family studied in *Ralstonia* have no LRR motifs.

There has been some confusion in the literature about the *Yersinia* YopM effector. This protein, like the other LRR-containing proteins mentioned above, belongs to the LPX effector family, which is characterized by the LPX (Leu-Pro-indefinite amino acid) motif, a subtype of the LRR motif [100]. A study by Soundararajan et al. reported the identification of an NEL domain towards the C-terminal tail of YopM [101]. However, the protein they studied appears to be a different protein with homology to the LRR family of effectors [102]. The conclusion is that YopM is an LRR protein that does not possess an NEL domain. Surprisingly, YopM has been described as an E3 ubiquitin ligase with a CLD (Cys68 Leu69 Asp70) catalytic motif in its N-terminal region [103]. However, this activity has not been confirmed in other studies and, since this protein lacks an NEL domain, it will not be further discussed in this review.

**Table 1:** Representative NEL T3SS effectors.

Organism	Hosts	Effector Name	Domains	References
*Salmonella enterica*	Mammals	SlrP	LRR-NEL	[104,105]
		SspH1	LRR-NEL	[106,107]
		SspH2	LRR-NEL	[106,108]
		SspH3	LRR-NEL	[109]
*Shigella flexneri*	Primates	IpaH proteins (1.4, 2.5, 4.5, 7.8, 9.8, a, b, c, d, e)	LRR-NEL	[107,110]
*Sinorhizobium fredii*	Plants	NopM	LRR-NEL	[111,112]
*Ralstonia solanacearum*	Plants	RipAR	NEL	[113]
		RipAW	NEL	[113]
		RipV1	NEL	[113,114,115]
		RipV2	NEL	[116]

**Figure 2:**
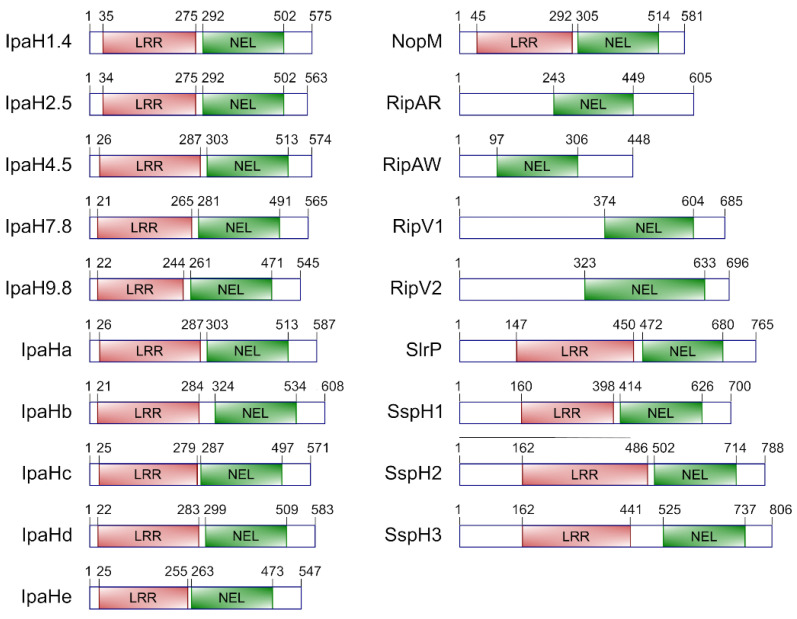
Diagram of domain structure of effectors of the NEL family of E3 ubiquitin ligases: IpaH proteins from *S. flexneri*; NopM from *S. fredii*; RipAR, RipAW, RipV1, and RipV2 from *R. solanacearum*; SlrP, SspH1, SspH2, and SspH3 from *S. enterica*. Boundaries for each domain are numbered according to the InterProScan predictions [99].

*Salmonella* leucine-rich repeat protein (SlrP) was identified in *S. enterica* serovar Typhimurium as a putative host range factor with homology to IpaH from *S. flexneri* [104]. The gene encoding this protein is located in a 2.9 kb chromosomal region that is outside the main pathogenicity islands but, because of its atypical G+C contents and its lack of conservation in other related bacteria, it is considered a pathogenicity islet. *Salmonella* secreted protein H1 (SspH1) was identified in another horizontally acquired DNA region [106] provided by prophage *Gifsy-3*, that is specifically present in strain 14028 of *S. enterica* serovar Typhimurium, but not in other representative strains of this serovar, such as LT2 or SL1344 [117]. The gene encoding SspH2 was identified by Southern blot analysis with *sspH1*-derived probes [106]. The three genes encoding these LPX-containing effectors exhibit a complex phylogenetic distribution among *Salmonella* serotypes [106]. Recently, a genomic analysis of a large collection of *Salmonella* genomes identified a gene known as *sspH3*, which encodes a protein, SspH3, that is 75% identical to SspH2. The same study detected the presence of *sspH1* in 11.9% of the 2544 whole-genome sequences analyzed, most of them belonging to serovars Typhimurium, Cerro and Javiana. In contrast, the prevalence of *sspH3* was 9.3%, with Agona, Tennessee and Kentucky as the leading serovars encoding this gene [109]. The domain structure of these effectors is shown in Figure 2.

*Shigella* possesses several invasion plasmid antigen h (*ipaH*) genes (Figure 2). The initial cloning of an *ipaH* gene from the virulence plasmid [118] was followed by the identification of five plasmid genes that were termed *ipaH1.4*, *ipaH2.5*, *ipaH4.5*, *ipaH7.8* and *ipaH9.8* based on the size in kb of the *Hin*dIII fragments where the genes were located [119]. In addition, the chromosome of different strains of *S. flexneri* contains five different *ipaH* homologous genes, some of which are duplicated in different strains [120]. These genes have been annotated with different names in different genomes. In strain 301, *ipaH0722* (*ipaH1*), *ipaH0887* (*ipaH2*), *ipaH1383* (*ipaH3*), *ipaH1880* (*ipaH4*), *ipaH2022* (*ipaH6*), *ipaH2202* (*ipaH5*) and *ipaH2610* (*ipaH7*) correspond to ORFs SF0722, SF0887, SF1383, SF1880, SF2022, SF2202 and SF2610, respectively [110]. Three of these genes, *ipaH0887*, *ipaH2022*, and *ipaH2202*, are considered pseudogenes in strains 301 and 2457T [121,122], although all appear to be functional in strain YSH6000 [110]. Here, we use the letter-based nomenclature and the protein sequences from strain 8401 where IpaHa, IpaHb, IpaHc, IpaHd and IpaHe correspond to IpaH0722, IpaH2610, IpaH1383, IpaH1880, and IpaH2022 in strain 301 [121]. The putative proteins encoded by these genes consist of four domains: an N-terminal secretion domain, an LRR region with a variable number of repeats, and intervening region, and the NEL C-terminal domain, which is almost identical in all of them.

NopM (Figure 2) was identified through a proteomic approach as a nodulation outer protein secreted via a T3SS in *S. fredii* HH103 [111]. It has a molecular weight of 64 kDa and is homologous to a protein initially named Y4fr in strain NGR234 [123]. Homologous sequences also exist in various rhizobial strains of the genus *Bradyrhizobium*.

*R. solanacearum* expresses at least four different T3SS effectors that are considered unusual members of the NEL family because they do not have LRR motifs (Figure 2). The gene encoding RipV1 was identified as gene *hpx29* in a genetic screen to find genes regulated by the HrpB transcriptional activator that controls the expression of most of the T3SS effector genes in these bacteria [124]. It was also named *rip12* in a screen that was carried out to determine the repertoire of effector proteins possessed by *R. solanacearum* RS1000 [114]. The protein was renamed RipV1 in a proposal for a unified nomenclature [115]. The same study defined RipV2 as an additional putative Rip protein through a phylogenetic analysis [115]. Finally, Rip61 and Rip69 were identified in the screen mentioned above [114] and renamed RipAR and RipAW, respectively [115].

**Figure 3:**
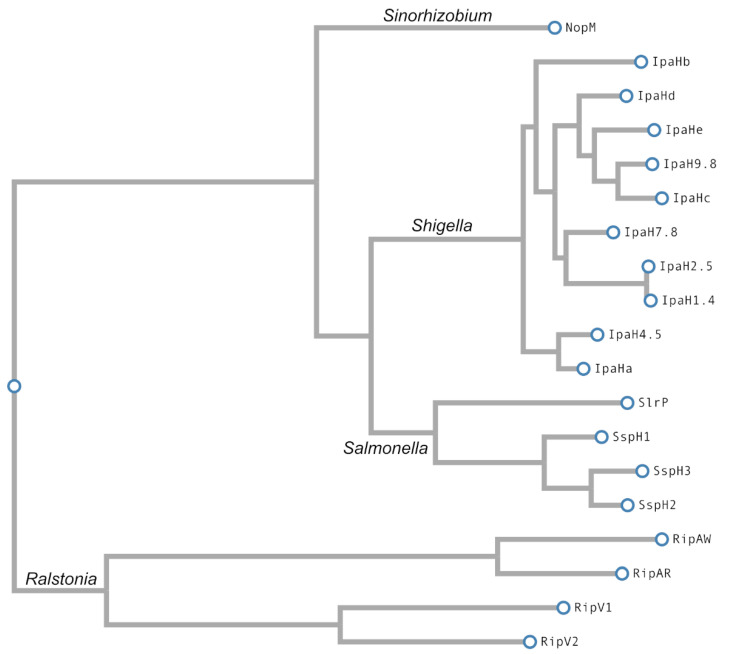
Phylogenetic analysis of NEL effectors. Alignment and phylogenetic reconstructions were performed using the function “build” of ETE3 v3.1.1 [125] as implemented on the GenomeNet (https://www.genome.jp/tools/ete/, accessed on 14 May 2022). Alignment was performed with Clustal Omega v1.2.1 with the default options [126]. The tree was constructed using FastTree v2.1.8 with default parameters [127].

A phylogenetic analysis of NEL effectors is shown in Figure 3.

### 3.2. Expression, Translocation, and Subcellular Localization

In this section, we explore the ways in which the expression of the genes encoding these effectors is coordinated with the expression of the T3SS involved in their secretion. We also summarize the conditions needed for their translocation and the subcellular localization in the host cells.

In *S. enterica*, SPI1 and SPI2, encoding T3SS1 and T3SS2, respectively, have very different patterns of expression and regulation. SPI1 is necessary for the first stages of infection, and its expression is directly controlled by the transcriptional activator HilA. The transcription of *hilA* is activated by HilC, HilD, and RtsA, three AraC-like proteins that form a regulatory feedforward loop [15,128]. These proteins act as antirepressors that counteract the effect of H-NS, a pleiotropic regulator that preferentially binds to AT-rich regions of the chromosome, that are typical of horizontally acquired genes in *Salmonella* [129]. Many environmental and physiological signals impact this regulatory system mainly through control of *hilD* mRNA translation or stability, or HilD protein activity. In addition, the promoter of *hilD* is the main target of the repressive effect of H-NS [129]. SPI2 is necessary for survival and replication within host cells. Its expression is induced in response to signals found in the SCV, where expression of SPI1 is repressed. The main direct transcriptional activator of SPI2 is the two-component system SsrA/SsrB [130]. There is a coordinated regulation of SPI1 and SPI2 that is mediated by several regulators. The PhoQ/PhoP two-component system is active under the conditions found inside the SCV, low pH and low Mg^++^ concentration. This system represses SPI1 expression [131] but activates SPI2 expression through activation of the SsrA/SsrB two-component system. Natural conditions that lead to optimal expression of T3SS1 or T3SS2 can be imitated in the laboratory using specific culture conditions. T3SS1 is optimally expressed in LB-rich medium with high osmolarity and microaerophilia, whereas T3SS2 is expressed in LPM minimal medium with acidic pH and low concentration of Mg^++^. Interestingly, T3SS2 can also be expressed in LB medium in a HilD-dependent manner, but whereas T3SS1 is expressed at the beginning of the stationary phase, the expression of T3SS2 is more evident towards the late stationary phase [132,133]. The four members of the NEL family described in *Salmonella* are encoded outside SPI1 and SPI2. Using the media described above to analyze *slrP* expression revealed that this gene is significantly expressed under SPI1- and SPI2-inducing conditions, although the highest expression was observed under the latter condition [134]. Lon and LeuO are negative regulators of *slrP* expression that act through HilD, while the two-component system PhoQ/PhoP directly activates *slrP* transcription under SPI2-inducing conditions. In vitro experiments suggested that translocation of the effector SlrP can occur through T3SS1, T3SS2, or both, depending on host cell type, timing, and specific incubation conditions. Similarly to SlrP, SspH1 is translocated by T3SS1 and T3SS2. However, expression of *sspH1* is maximal under conditions for optimal expression of SPI1, and this gene is not induced in intracellular bacteria. In contrast, *sspH2* is induced in intracellular bacteria in a SsrA/SsrB-dependent manner, and its product, SspH2, is specifically translocated through T3SS2 [106]. Another study confirmed positive transcriptional regulation by SsrB of *sspH2* but not of *slrP* [135]. These three *Salmonella* NEL effectors share a similar amino-terminal region of about 142 amino acids that is responsible for their secretion through a T3SS. This translocation domain is also found in other effectors with different functions, including SifA, SifB, SseI, and SseJ [136]. In addition, SspH3, a recently identified *Salmonella* member of the NEL family, also has an N-terminal domain that is 77% similar to the translocation domain in SspH2. SspH3 heterologously expressed from a plasmid has been shown to be secreted to the culture supernatants in a T3SS1-dependent manner [109]. Translocation into host cells or T3SS2 secretion have not been investigated for this effector yet. The subcellular localization in the host cell has been studied for SlrP, SspH1, and SspH2. SlrP was evenly distributed in the cytoplasm of mouse macrophage-like Raw-TT10 and CHO-K1 cells after transient transfection [137]. Fluorescent microscopy and subcellular fractionation also suggest that a significant amount of SlrP is directed to the endoplasmic reticulum [138]. SspH1 was predominantly found in the nucleus after translocation into human Intestine-407 cells or transient transfection into Raw-TT10 or CHO-K1 cells [137]. In contrast, SspH2 was localized to the plasma membrane and was enriched in areas of actin polymerization after transfection [108,139], and this localization was dependent on the S-palmitoylation of a conserved cysteine residue within its N-terminal domain [140].

The expression of the T3SS in *Shigella* is regulated by one general repressor, H-NS, and three specific transcriptional activators, VirF, VirB and MxiE [20,141]. As in *Salmonella*, H-NS silences expression of virulence genes by preferentially binding to AT-rich regions, like those found in T3SS genes, that have an AT content that is higher than 60% [142]. One important regulatory environmental factor is temperature. At 30 °C, H-NS binding represses the expression of several genes located in the virulence plasmid: regulatory genes *virF* and *virB*, as well as genes encoding structural parts of the T3S apparatus and several substrates [20,141]. Shifting the temperature to 37 °C, the human body temperature, relieves H-NS binding to the promoter of *virF*. Other signals sensed by *virF* are pH, through the two-component system CpxA/CpxR, and osmolarity [143]. A combination of appropriate signals would then allow the production of the VirF protein, an AraC-like regulator that directly activates the transcription of *icsA* and *virB*. The product of *icsA* is essential for cell-to-cell spread [144], whereas *virB* encodes a second transcription activator, VirB [145]. This protein activates the transcription of the genes encoding the T3SS (Mxi and Spa proteins), its early effectors and their chaperones (Ipa and Ipg proteins) [143]. VirB also activates transcription of the gene coding for MxiE, another AraC-like protein that is the last regulator in the cascade. This regulator, helped by the IpgC chaperone, is necessary for the expression of late T3SS effectors [146]. According to their expression profiles, *Shigella* T3SS substrates can be classified into three categories: (i) effectors controlled by VirB, (ii) effectors controlled by MxiE, and (iii) effectors controlled by both VirB and MxiE [147]. The *ipaH* genes belong to the second category: five are scattered throughout the virulence plasmid, outside the entry region, and the others are in the chromosome, but all of them have a conserved MxiE box in their promoter region with the consensus sequence 5′-GTATCGTTTTTTTAnAG-3′ (where n represents a non-conserved nucleotide) [120]. The IpaH proteins are second-wave effectors that are translocated only after the shift of T3SS to its active state upon contact with host cells (or treatment with Congo red) and secretion of prestored translocators and first-wave effectors [148]. Although the IpaH proteins are very similar to each other, they differ in subcellular localizations. An early study concluded that IpaH9.8 is secreted from intracellular bacteria and transported into the nucleus in infected HeLa cells. This nuclear localization was confirmed in transfected COS-7 cells [149]. Because of their nuclear localization, IpaH9.8 and *Salmonella* SspH1 (together with effectors from other families) are considered nucleomodulins: bacterial factors acting within the nucleus on gene expression or other nuclear processes [150]. In contrast, IpaH7.8 was found in the cytoplasm of transfected J774A.1 macrophages [151], whereas IpaH0722 (IpaHa) localized to the cell membrane of Cos-7 and HeLa cells and palmitoylation at Cys14 and Cys18 residues is critical for this localization [152]. Constitutive secretion into the culture supernatant can be achieved in an *ipaB ipaD* mutant. Under these conditions, secretion of IpaH1.4, IpaH7.8, IpaH9.8, IpaHb, and IpaHe was detected in a proteomic study [153].

Most T3SS genes of *S. fredii* are organized in three major genetic units [154]. One of these units is a highly conserved operon that contains most of the structural genes of the system. Expression of these genes is co-regulated with expression of *nod* genes (genes involved in nodulation) and responds to plant-made flavonoids. These are secondary metabolites that interact with NodD proteins [155,156] of the LysR family of transcriptional regulators that then bind to *nod* box regulatory sequences [157] located in the promoters of *nod* genes and the gene *ttsI* (previously known as y4xI). In *S. fredii* HH103, NodD1 is a positive regulator of *ttsI*, while NodD2 has no effect on its transcription and NolR acts as a repressor [158]. The product of *ttsI* is a transcriptional activator, TtsI, which is the main regulator of the expression of T3SS genes [158,159]. TtsI binds to *tts* boxes, with the consensus sequence 5′-TCGTCAGCTTnTCGAAAGCTnnnCCnCnTA-3′ [159,160], that are found in the promoter regions of T3SS-related genes [161]. The NEL family member NopM is one of the T3SS effectors whose expression is induced by flavonoids [111]. Secretion of NopM by the T3SS to culture supernatants was also detected in strain NGR234 [123], and translocation into plant cells was demonstrated using a *Xanthomonas*/pepper translocation system [162]. The promoter of *nopM* (y4fR) contains a conserved *tss* box [159], and its activity depends on TssI [161]. NopM was first described as a T3SS-secreted protein in strain HH103 using a proteomic approach [111]. A study using a bimolecular fluorescence complementation method suggested dimerization of NopM at plasma membranes in onion cells, whereas expression of GFP- or RFP-NopM fusions showed fluorescence in the whole cell [112].

The *hrp* genes encoding structural components, regulators, and some effectors of *R. solanacearum* T3SS are in a cluster with seven transcription units located in the megaplasmid, whereas most effector genes are scattered around the genome [30,163]. Transcription of these genes is directly controlled by HrpB, a transcriptional regulator belonging to the AraC family that binds to a conserved plant inducible promoter (PIP) motif (consensus 5′-TTCGC-n15-TTCGC-3′) [124], also known as *hrp_II_* box (5′-TTCG-n16-TTCG-3′) [164]. The transcription of *hrpB* is activated by two similar proteins, HrpG and PrhG, that belong to the OmpR/PhoB family of two-component response regulators. HrpG integrates three major signals: physical contact with the plant host, bacterial growth conditions, and a quorum sensing signal that is transduced by PhcA, a LysR transcriptional regulator that responds to cell density and negatively regulates *hrpG* expression [165]. Bacterial contact with plant cells is sensed through PrhA [166], a protein homologous to outer membrane siderophore receptors [167] that initiates the regulatory cascade PrhA-PrhR/PrhI-PrhJ-HrpG [168]. Expression of *hrpG* is also induced by a cell contact-independent and PrhA-independent signal of unknown nature [169]. The expression pattern of *prhG*, the alternative transcriptional activator of *hrpB*, is totally different from that of *hrpG*. It is induced after growth of bacteria in minimal medium but not in the presence of host cells [170]. In addition, *prhG* is activated by PhcA, suggesting a switch from HrpG to PrhG to ensure *hrpB* activation at different cell densities [171]. Two additional positive regulators of *hrpB* that act through *prhG* are CysB, which is necessary for induction in nutrient-rich and cysteine-supplemented minimal media [172], and PrhN, a MarR positive regulator whose expression increases with cell density [173]. The LysR-type transcriptional regulator PrhO positively regulates the expression of both *hrpG* and *prhG*, although this regulation is not mediated by PrhJ, PhcA, or PrhN, and the direct targets of PrhO are unknown [174]. Another example of T3SS regulators is the PadR-like protein PrhP that is necessary for full expression of *hrpB* in *hrp*-inducing medium in a *hrpG*- and *prhG*-independent manner [175]. The gene encoding the NEL effector RipV1 was identified as HrpB-regulated in a genetic screen using a transposon-based system [124]. A PIP box was found in the promoter of this gene. Another study also identified a PIP box in the promoter of *ripAR* and showed *hrpB*-dependent expression for this gene. In contrast, no PIP box motif was found in the upstream region of *ripAW*, and its expression was very low and *hrpB*-independent [114]. The same study showed T3SS-dependent translocation into plant cells for RipV1, RipAR and RipAW in a functional screen based on a *Bordetella pertussis* calmodulin-dependent adenylate cyclase (Cya) reporter. Translocation of the three effectors required HpaB [114], a class IB chaperone that was shown to interact with RipAW in a yeast two-hybrid assay and in GST pull-down experiments [176]. Transient expression of GFP fusions in *Nicotiana benthamiana* protoplasts indicated that RipAR and RipAW localized to the cytoplasm of protoplasts [113]. A similar procedure showed that RipV2 localized to the plasma membrane [116].

It is interesting to note that although these effectors are typically delivered to host cells through a T3SS, several LPX effector proteins, including IpaH1.4, IpaH2.5, IpaH7.8 IpaH9.8 and SlrP, have been shown to be able to enter host cells in a T3SS-independent manner and are considered cell-penetrating effectors. Specifically, IpaH9.8 was internalized via macropinocytosis and lipid raft-dependent endocytosis, followed by endosomal escape [177].

### 3.3. Enzymatic Activity and Structural Studies

As mentioned above, NEL domains display a particular structure that differs from the canonical RING, HECT and RBR families, allowing, therefore, the grouping of NEL effectors in a new family of E3 ligases. We summarize here structural and functional studies that help to understand the mechanism of inhibition and activation provided by the interaction between the two modules that are present in the typical members of the family.

The first study demonstrating the enzymatic activity of these effectors reported that IpaH9.8 from *Shigella* and SspH1 from *Salmonella* constitute a new class of E3 ligases [107]. The term NEL, from “Novel E3 ligase”, was introduced in a publication that describes the crystal structure of the *Salmonella* T3SS effector SspH2 [108]. This and other crystallographic reports revealed that the overall structure of typical effectors of the NEL family is composed of two distinct modules: a banana-shaped LRR domain that is structurally similar to the *Yersinia* effector YopM, and a C-terminal catalytic domain that contains a number of α-helices displaying a unique α-helical fold [56,57,108,178,179]. These extensive biochemical studies on crystal structures of NEL effectors confirmed that they represent a new class of E3 ligases, found exclusively in pathogenic or symbiotic bacteria, which are structurally and mechanistically distinct from canonical RING/HECT ubiquitin ligases. The LRR module is involved in substrate recognition [180], and the variable number of LRR motifs in different NEL effectors may be related to substrate specificity binding, while the NEL domain exhibits the ubiquitination ligase activity. Both domains are bridged by a ~10 aa hinge loop [56] that provides flexibility to the molecule. This flexibility is crucial for the autoregulation of the NEL effectors (see below).

Similarly to HECT E3 ligases, NEL effectors contain a conserved cysteine residue that is essential for the enzymatic activity. This cysteine residue is within a conserved CXD motif that acts as a catalytic nucleophile in the ubiquitination reaction [56,107,108,179,181]. Both types of ligases catalyze ubiquitin transfer from the corresponding E2 conjugating enzyme to catalytic cysteine through a transthiolation reaction and then to a specific substrate after nucleophilic attack of the E3-ubiquitin thioester bond by a lysine side chain of the target protein [182]. In addition to the catalytic cysteine, other conserved residues located near the catalytic center have been shown to be important for the enzymatic activity, such as the Asp339 and Asp397 residues of IpaH9.8. These charged residues are suggested to maintain a favorable electrostatic environment for the reaction [57,183]. In addition, in IpaH3 (IpaHc/IpaH1383), the Asp365 residue within the CXD active center is important for the enzymatic activity. Enzymes mutated on this Asp residue are unable to catalyze the polyubiquitination reaction, while keeping the capacity to hydrolyze E2-ubiquitin intermediates, suggesting that this conserved residue is involved in the transfer of ubiquitin to a lysine residue of the substrate [56]. Several NEL effectors, including IpaHc, SspH2, or IpaH9.8, have been shown to preferentially catalyze the synthesis of K48-linked polyubiquitin chains and to use UbcH5~ubiquitin as preferential E2 [56,58].

**Figure 4:**
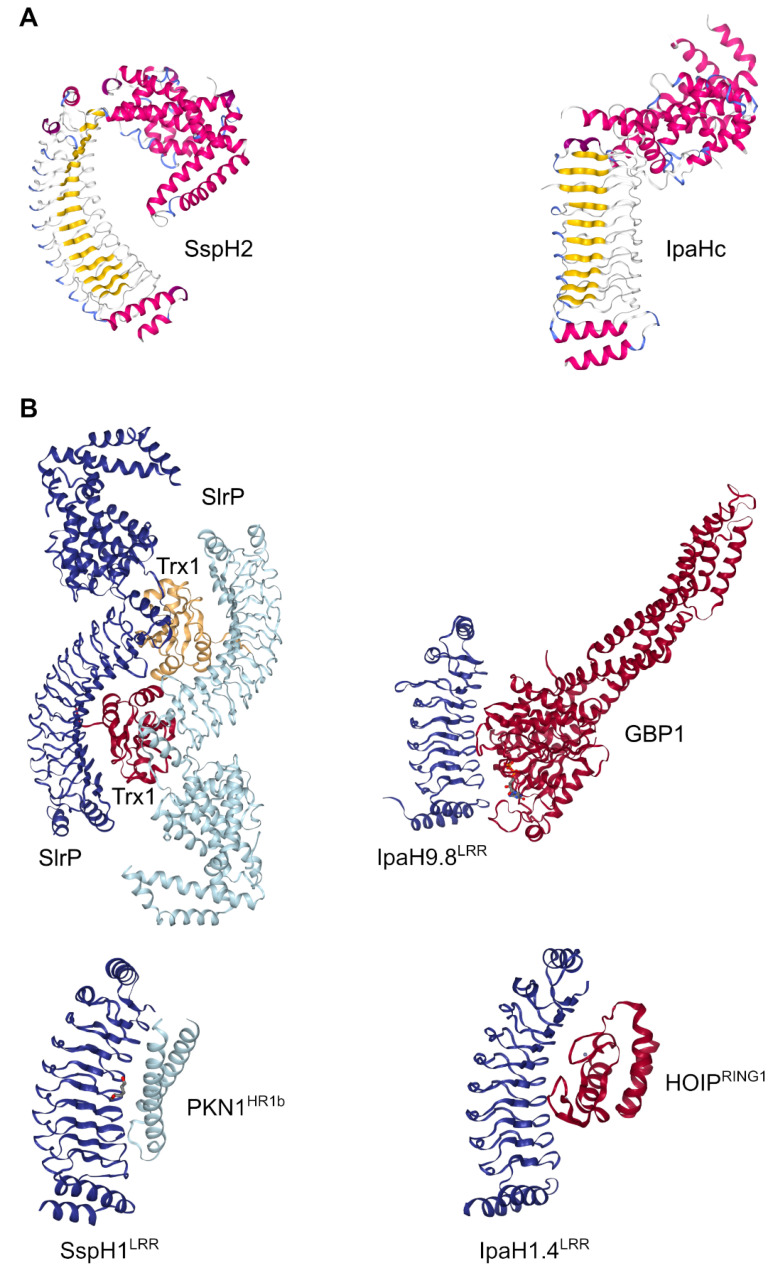
Crystal structures of NEL E3 ligases. (**A**) Examples of the two autoinhibition modes: SspH2, mode 1 (PDB ID: 3G06) [108]; IpaHc, mode2 (PDB ID: 3CVR) [56]. Colors are set according to secondary structure. (**B**) E3 ligases in complex with their substrates: SlrP (blue) in complex with Trx1 (red and yellow) (PDB ID: 4PUF) [179]; the LRR domain of SspH1 (dark blue) in complex with the HR1b domain of PKN1 (light blue) (4NKG) [186]; the LRR domain of IpaH9.8 (blue) in complex with GBP1 (red) (PDB ID: 6LOJ) [185]; and the LRR domain of IpaH1.4 (blue) in complex with the RING1 domain of HOIP (red) (PDB ID: 7V8G) [187]. Drawings created with NGL [191].

The crystal structures of the NEL effectors mentioned above revealed an autoregulated mechanism that could prevent premature ubiquitination activity [56,57,108] that would lead to unwanted degradation by the host ubiquitination system [184]. Autoinhibited NEL enzymes are not completely inactive, but maintain the capacity to bind ubiquitin from E2 [59]. According to these structures, the LRR domain folds to interact with the NEL domain, blocking the catalytic cysteine residue and inhibiting the enzymatic activity. Two distinct autoinhibition modes [185] have been proposed based on the structures of SspH2 (mode 1) [108] and IpaHc (mode 2) [56]. In mode 1, the concave surface of the LRR domain is oriented toward the NEL domain, whereas in mode 2, the concave surface is oriented in an opposite direction relative to the NEL domain (Figure 4A). For both autoinhibition modes, the activation mechanism involves the substrate binding to the LRR domain, which would induce a drastic conformational change between the two modules that would lead to the release of the catalytic cysteine [108,184]. The mechanistic details to relieve autoinhibition in mode 1 were provided by other structural studies, based on substrate-bound complexes, such as SspH1^LRR^-PKN1^HR1b^ [186] or SlrP-Trx1 [179]. More recently, the crystal structure of the IpaH9.8^LRR^-hGBP1 complex revealed that the C-terminal hydrophobic pocket of LRR domain is important for the interaction with the NEL domain in mode 2. Further analysis showed that Arg166 and Phe187 function as sensors for substrate interaction [185].

In addition, the complexes between the LRR domain of IpaH1.4 and two of its binding partners have recently been determined [187]. In all of these complexes, the ligases bind their targets using the concave surface of their LRR domains [188] (Figure 4B). The structure of SlrP-Trx1, however, suggests that the linker region between the NEL and LRR domains plays an essential role in substrate binding [179]. Another difference between members of the family concerns the formation of dimers. Constructs of IpaH9.8 or SlrP containing both LRR and NEL domains form dimers either alone or bound to the substrate [179,189], whereas SspH1 is monomeric [190].

Autoinhibition has also been suggested for the *S. fredii* effector NopM, since enzyme activity of the full-length protein was lower than that of the NEL domain alone in an in vitro ubiquitination system [112]. Although no host target was identified, the same study showed that NopM was able to form free K48-dependent polyubiquitin chains and to ubiquitinate itself.

### 3.4. Role in Virulence or Symbiosis

The group of pathogenic bacteria discussed here expresses a set of translocated T3SS effectors that exert a complex combination of effects on the host defense response. Thus, the specific physiological role of an individual effector in the context of infection and its contribution to virulence is often difficult to determine, especially in a case like this, where there may be several effectors of the same family expressed simultaneously in the same bacterium. Four effectors of the NEL family have been described in *Salmonella* and *Ralstonia*, and up to ten IpaH effectors in *Shigella*. A certain degree of redundancy may explain why single effector mutations have moderate or no effect on virulence.

In *S. enterica* serovar Typhimurium, an early study showed that SlrP was necessary for full virulence in mice but not in the bovine host, and hence, was considered a host range factor. During competitive infection experiments, the *slrP* mutant was recovered in significantly lower numbers than the wild type from murine but not bovine Peyer’s patches, and this mutant was attenuated sixfold in mice but was as virulent as the wild type in calves [104]. Another study showed that mutations in *slrP* or *sspH1* did not reduce the ability of *Salmonella* to elicit fluid secretion in bovine ligated ileal loops [192]. Strains with single mutations in *sspH1* or *sspH2* are not attenuated in calves, while a double *sspH1 sspH2* mutant strain is attenuated in this model [106], suggesting some redundancy between effectors. However, deletion of *sspH2* reduced by 30-fold the virulence of *S. enterica* serovar Enteritidis in BALB/c mice [193]. In addition, a mouse model (strain 129SvJ) to study the role of single effectors in persistence, the ability of bacteria to survive in the host weeks after infection, revealed that both SspH1 and SspH2 significantly contributed to persistence of *S. enterica* serovar Typhimurium during systemic infection [194]. Studies carried out at the cellular level suggested that heterologous expression of SlrP in human epithelial HeLa cells induced defects in migration and adhesion [195] and increased cell death [105]. Additionally, using HeLa cells as infection model, SspH1 was shown, together with SptP, to downregulate the NF-κB-dependent expression of the pro-inflammatory cytokine IL-8 [137]. In contrast, SspH2 increased Nod1-dependent IL-8 production in infected epithelial cells [196]. Early experiments showed that SspH2 inhibited the rate of actin polymerization in vitro; however, an *sspH2* mutant of *S. enterica* serovar Typhimurium was not impaired in the ability to form vacuole-associated actin polymerizations [139]. Interestingly, SlrP and SspH2 are important factors contributing to the ability of *Salmonella* to inhibit stimulation of antigen-dependent T-cell proliferation by bone marrow-derived dendritic cells [197]. Another study also identified these effectors as inhibitors of migration of dendritic cells toward the chemokine CCL19 [198].

Although shigellosis is an intestinal disease, intestinal infection caused by *Shigella* cannot be easily studied since this human-restricted pathogen does not infect mice orally. Therefore, in addition to cellular infection models, two alternative animal models have been used to test *Shigella* virulence: the Sereny test and a murine lung infection model. In the Sereny test, a bacterial suspension is inoculated into the conjunctivae of guinea pigs, and the animals’ eyes are examined for several days for evidence of keratoconjunctivitis [199]. The murine lung infection model is a well-established model for studying *Shigella* pathogenesis that reproduces both the inflammatory response and the bacterial clearance observed in dysentery [200]. An early study showed that mutants with deletions of plasmid genes *ipaH4.5* and *ipH7.8*, alone or in combination, displayed normal rates of HeLa cell invasion [201], suggesting that these effectors do not participate in the initial steps of the infection. However, these mutants caused a significantly exacerbated Sereny reaction in guinea pig eyes, indicating that they play a role in downmodulation of the inflammatory response. In addition, deletion of *ipaH7.8*, but not *ipaH4.5* or *ipaH9.8*, resulted in a dramatic increase in recovery of colony forming units, similarly to those observed upon infection with plasmid-cured avirulent strains, after infection of murine J774 macrophages, and these mutant bacteria were predominantly contained within the endocytic vacuoles, suggesting a role for IpaH7.8 to facilitate the escape of virulent bacteria from the vacuole [201]. In contrast, another study showed that *ipaH7.8* mutants were able to escape the phagosome in RawB murine macrophages, but these mutants were less cytotoxic than the wild-type strain [202]. The role of IpaH7.8, as well as IpaH4.5, in potentiating murine macrophage killing was confirmed by other authors [151,203]. However, IpaH7.8 appears to prevent pyroptosis in human endothelial cells [204], and IpaH1.4 was shown to inhibit *Shigella*-triggered epithelial cell death [205]. In the murine lung infection model, an *ipaH9.8* mutant caused more severe inflammatory responses with enhanced pro-inflammatory cytokine secretion levels in comparison with the wild-type strain, leading to a 30-fold bacterial colonization reduction [206], pointing to the possibility that IpaH9.8 participates in the attenuation of acute inflammatory host responses. Similar results were observed using an *ipaH4.5* mutant, which also elicited a more severe inflammatory response than the wild-type strain in both the Sereny test and a murine lung infection model [207]. This study also revealed that deletion of *ipaH4.5* did not affect the invasion of epithelial cells and macrophages by *S. flexneri* when assayed in vitro. Another study confirmed a contribution of IpaH4.5 to pathogenesis in the murine lung infection model and revealed a role of this effector in subverting antigen cross-presentation to cytotoxic T lymphocytes [208]. The potential role in virulence of chromosome-encoded IpaH effectors (IpaHa-e) was also investigated [110]. Although single mutations did not result in any appreciable effect, a mutant lacking all of the chromosomal *ipaH* genes (but keeping the plasmid *ipaH* intact) induced an exacerbated inflammatory response in a mouse pulmonary infection model and attenuated mouse lethality.

The role in virulence of NEL effectors of the plant pathogen *R. solanacearum* has been less explored than in the case of animal pathogens. However, three of these effectors, RipAR, RipAW, and RipV2, were shown to suppress plant PTI responses, such as the production of reactive oxygen species and the expression of defense-related genes when expressed in leaves of *N. benthamiana* [113,116]. These effects were mediated by their E3 ubiquitin ligase activity. Notably, RipAW also triggers ETI and induces cell death in *N. benthamiana* and *N. tabacum* [209]. Although the double mutant *ripAR ripAW* was not attenuated in tomato or pepper plants, transgenic *Arabidopsis* plants expressing *ripAR* and *ripAW* were more susceptible than wild-type plants to infection with *P. syringae*, suggesting a contribution of RipAR and RipAW to the virulence of *R. solanacearum* in certain plants [113]. Interestingly, *ripV2* mutants showed obvious disease attenuation compared with the wild-type strain in pathogenicity assays on potato [116]. Indeed, in a previous study, *ripV2* was identified as a virulence-associated gene in tomato [210].

Comparably to *Ralstonia* NEL effectors, NopM from *S. fredii* strain NGR234 induced cell death [112] and inhibited production of reactive oxygen species in tobacco [211]. Rhizobial T3SS effectors may promote or inhibit symbiosis depending on the host [212]. In the case of *S. fredii*, studies comparing wild-type and *nopM* mutant strains revealed that NopM promotes symbiosis in the interaction with the host plant *Lablab purpureus* [123], and this positive effect depends on its E3 ubiquitin ligase activity [211]. In contrast, NopM has a slight negative effect in the interaction with *Pachyrrizus tuberosus* and *Crotalaria juncea* [123] and no effect in the interaction with *Phaseolus vulgaris* or *Flemingia congesta* [211]. In addition, heterologous expression of NopM diminished nodulation in *Lotus japonicus* [112].

### 3.5. Targets of NEL Effectors

It is usual that bacterially delivered effectors target host cell ligands to tackle cellular processes. The identification of these host ligands is a crucial step to understand how pathogens modulate host signaling pathways to their own benefit. The differential subcellular location in the host described above for effectors of the same family secreted by the same bacteria may be one of the factors contributing to confer specificity to the interaction with particular substrates. Here, we summarize identified host substrates for *Salmonella* and *Shigella* NEL E3-ubiquitin ligase effectors (Table 2). No host targets have been identified yet for members of the family from *Sinorhizobium* or *Ralstonia*.

In the nucleus, the *Salmonella* effector SspH1 interacts with protein kinase N1 (PKN1) [180], a serine/threonine kinase of the protein kinase C (PKC) superfamily involved in the regulation of several pathways related to host immune signaling. The interaction occurs through the LRR domain of SspH1 [180] and the HR1b coiled-coil subdomain of PKN1 [186]. PKN1 is a positive regulator of androgen receptor, mineralocorticoid receptor, and progesterone receptor signaling, and a negative regulator of Akt and NF-κB signaling. SspH1 catalyzes the ubiquitination of PKN1 in vitro [107] and in vivo, leading to its proteasome-dependent degradation and resulting in the attenuation of the androgen receptor response [186]. However, the NF-κB suppression induced by SspH1 [137] appears to be independent of its catalytic activity and its ability to bind PKN1, suggesting that other cellular targets may be mediating this effect [186].

SspH2 was first reported to interact with the actin binding proteins filamin and profilin (PFN1) by a yeast two-hybrid screen, also showing that SspH2 inhibits the rate of actin polymerization in vitro. Whereas the filamin-binding domain was located in the amino-terminal 61 residues of SspH2, the carboxy-terminal domain of the effector was necessary for the interaction with profilin [139]. Interestingly, a global ubiquitinome analysis, carried out in the context of *S. enterica* serovar Typhimurium-infected cells, identified the cytoskeleton-related proteins ACTR8, ARPC5, FLNA, MYH9, SUGT1, PFN2 and DYNC1H1 as potential host targets for SspH2 [213]. In addition, MYH9 was also identified as a target for SspH2 and SspH1 in another quantitative proteomic analysis [213]. However, further studies are needed to confirm these interactions and to investigate whether these host ligands are substrates of the SspH2 E3 ligase activity. A quantitative mass spectrometry technique used after immunoprecipitation of SspH2 expressed in human HEK293T cells confirmed the interaction with SGT1 (also known as SUGT1) and identified four additional binding partners: AH receptor interacting protein (AIP), Bub3, 14-3-3γ, and Bcl-2-associated athanogene regulator 2 (BAG-2) [214], although only the interaction with SGT1 has been investigated in detail [196]. Mammalian SGT1 is required for progression through G1/S and G2/M checkpoints of the cell cycle and has a second function in innate immunity as an NLR co-chaperone. SGT1 is a co-chaperone for Nod1, a canonical NLR that signals through NF-κB to express pro-inflammatory chemokines such as IL-8. Interestingly, SspH2 bound NLR co-chaperone-competent SGT1 and Nod1 in a trimeric complex, monoubiquitinated Nod1, and caused agonist-independent activation of Nod1 [196]. A recent report used the viral-like particle trapping technology Virotrap and mass spectrometry to identify SspH2 interactors [215]. Virotrap confirmed interaction with PFN1 and PFN2, but interaction with SGT1 was not conclusive, and Nod1 was not detected, presumably due to the low level of expression of this protein in available cell lines.

SlrP interacts with thioredoxin and catalyzes its ubiquitination, affecting its reducing activity and leading to host cell death [105]. SlrP also binds ERdj3, an endoplasmic reticulum luminal chaperone, and interferes with the folding activity of this protein, leading to the accumulation of unfolding substrates in this organelle, which can also contribute to host cell death [138].

IpaH effectors from *S. flexneri*, once translocated to the host cell, target and ubiquitinate specific host ligands involved in mounting a proper innate inflammatory response. Several works provide evidence that IpaH9.8 downregulates or modulates the NF-κB mediated inflammatory response by direct targeting and ubiquitination of host proteins [216,217,218]. The first demonstration of the E3 ubiquitin ligase activity of the IpaH effectors showed that IpaH9.8 impairs the MAPK-dependent signaling in a yeast surrogate model by ubiquitin-dependent degradation of Ste7, a MAPKK involved in the pheromone response route [107]. In a more physiological context, IpaH9.8 recruits and ubiquitinates the splicing factor U2AF^35^, downregulating the expression of pro-inflammatory cytokines such as IL-8 [206,217]. Another identified target for IpaH9.8 is NEMO. IpaH9.8 interacts with NEMO/IKKγ and the ubiquitin-binding adaptor protein ABIN-1, leading to polyubiquitination and proteasomal degradation of NEMO, which in turns alters NF-κB activation [218]. IpaH9.8 also ubiquitinates GBP1, GBP2, and GBP4, members of the interferon-induced GTPase family of guanylate-binding proteins (GBPs), to prevent coating of *Shigella* with these proteins, which would lead to inhibition of actin-dependent motility and cell-to-cell spread of bacteria [216,219,220].

Another *Shigella* member of the family, IpaH0722 (IpaHa), binds and mediates ubiquitination and proteasome-dependent degradation of TRAF2 [152]. This appears to be a mechanism that downregulates the acute inflammatory response by blocking activation of the PKC-NF-κB pathway during invasion of epithelial cells.

IpaH4.5 directs ubiquitination of p65, one of the subunits of NF-κB, and inhibits this signaling pathway [207]. This effector also polyubiquitinates TANK-binding kinase 1 (TBK1) and promotes its proteasome-dependent degradation, preventing the phosphorylation, nuclear translocation, and activation of IFN regulatory factor 3 (IRF3) and the subsequent activation of innate immunity pathways [221]. In addition, IpaH4.5 targets the proteasome regulatory particle non-ATPase 13 (RPN13) and induces its degradation. RPN13 degradation disrupts proteasome-catalyzed peptide splicing and reduces antigen cross-presentation to CD8+ T cells via MHC class I in vitro, suggesting that this is a *Shigella* strategy to dampen the antigen-specific cytotoxic T lymphocyte response [208]. The fate of ubiquitinated targets is not necessarily degradation. An example is NLRP3, which is stabilized after binding to IpaH4.5 and K63 polyubiquitination, and this stabilization leads to inflammasome activation and pyroptotic cell death in macrophages [203]. Together, these results indicate that this effector can have opposite effects on the modulation of inflammasome activation depending on specific targets, cell types, and infection steps. Unexpectedly, IpaH4.5 has also been reported to possess a TBC-like GTPase-activating protein (GAP) activity towards the Rab GTPase Rab31 that would attenuate lysosomal function [222].

Two additional members of the IpaH family, IpaH1.4 and IpaH2.5, inactivate the NF-κB inflammatory pathway by direct interaction with HOIL-1L and HOIP subunits of linear ubiquitin chain assembly complex (LUBAC) and K84-linked ubiquitination followed by proteasomal degradation of HOIP, an E3 ligase of the RBR family [223]. Recruitment of LUBAC to activated cytokine receptors or pattern recognition receptors initiates an inflammatory response by binding of M1-linked linear ubiquitin chains to RIPK1 and NEMO (IKKγ). This positively regulates the NF-κB, allowing the expression of cytokines and mounting the inflammatory response [224,225,226]. This study suggested that, through the inactivation of the LUBAC machinery, IpaH1.4 and IpaH2.5 collaborate with other effectors in the suppression of immune receptor signaling [223]. However, in contrast with previous results, the same study showed no effect of IpaH4.5, IpaH9.8 or IpaH0722 (IpaHa) on the stability of p65, NEMO and TRAF2. In addition to NEMO, LUBAC-synthesized M1-linked ubiquitin chains also recruit optineurin, and both NEMO and optineurin protect cells (murine embryonic fibroblast were used in this study) against proliferation of bacteria such as *S. enterica* serovar Typhimurium in the cytosol. In contrast, *S. flexneri* are professional cytosol-dwelling bacteria that use IpaH1.4 to antagonize this pathway [227]. Interestingly, a recent report suggested that in addition to inducing the proteasomal degradation of LUBAC, IpaH1.4 can also inhibit the ubiquitin ligase activity of LUBAC by blocking its E2 loading and/or disturbing its stability [187].

IpaH7.8 is another NEL E3 ligase that promotes rapid macrophage cell death through the activation of NLR inflammasomes. This effect is mediated by polyubiquitination and degradation of glomulin (GLMN), a member of the S-phase kinase-associated protein 1–F-box–like complex, promoting cell death [151]. GLMN binds cellular inhibitor of apoptosis proteins 1 and 2 (cIAP1 and cIAP2), members of a family of RING-E3 ligases, reducing E3 ligase activity and inflammasome-mediated death of macrophages. In this context, the IpaH7.8-GLMN-cIAPs axis is a mechanism by which *Shigella* can accelerate macrophage pyroptosis, causing severe inflammation [228]. A second role has been recently proposed for IpaH7.8 during infection: suppression of NK-cell-mediated killing of *S. flexneri* by ubiquitinating and targeting gasdermin B (GSDMB) for proteolytic destruction. GSDMB belongs to a large family of pore-forming cytolysins, and this study showed a specific targeting of this protein and no other members of the family by IpaH7.8 [229]. This substrate of IpaH7.8 was identified using a technique termed ubiquitin-activated interaction traps (UBAIT) [230], which also confirmed the interaction of IpaH2.5 with HOIP and other members of the TNF receptor signaling complex, and the reaction of IpaH9.8 with GBP1, GBP2 and GBP4 from lysates prepared from interferon gamma-stimulated Caco-2 cells [230]. Another study demonstrated that IpaH7.8 can also ubiquitinate and induce the degradation of the pore-forming protein gasdermin D (GSDMD), preventing pyroptosis in human but not murine cells, which may explain the different susceptibility of humans and mice to *Shigella* infection [204]. The N-terminal pore-forming domain of GSDMD was required for IpaH7.8-induced degradation, and the addition of an N-terminal tag prevented degradation, which may explain why this target was not identified in the study mentioned above [229].

## 4. Conclusions and Perspectives

Ubiquitination is a post-translational modification that controls most cellular processes in eukaryotic cells. Therefore, although ubiquitin is absent in bacteria, it is not surprising that many pathogenic and symbiotic bacteria exploit this process to manipulate the host by injecting enzymes into eukaryotic cells that modify ubiquitination. The NEL family of E3 ubiquitin ligases is especially interesting in this context, since these are completely novel enzymes, with structural differences compared to mammalian E3 enzymes. NEL proteins have been characterized in a few bacterial species, but analysis of sequences in databases predicts that more than 900 species encode proteins with the NEL domain.

Although some host protein targets for NEL E3 ligases have been described, many more are expected to exist. In particular, no substrate has been described yet for members of the family found in plant pathogens and symbionts. The definition of these targets would be an important step to fully understand the function of these effectors. Different methods that are being developed to analyze the whole cellular ubiquitinome are of great interest to achieve this goal. These methods rely on mass spectrometry analysis after enrichment with affinity approaches that include the use of anti-ubiquitin antibodies, ubiquitin-binding proteins, or epitope-tagged ubiquitin [231]. An improvement has been the use of an antibody that recognizes diglycine-containing isopeptides after trypsin digestion, as trypsinolysis of ubiquitin conjugates yields a characteristic diGly remnant [232]. UbiSite is another antibody-dependent technology that recognizes the C-terminal 13 amino acids of ubiquitin, which remain attached to modified peptides after proteolytic digestion with the endoproteinase LysC [233]. UbiSite discriminates ubiquitin from other Ubl proteins (NEDD8 or SUMO) and allows mapping of modified lysines of the substrates. A complementary alternative is the ubiquitin-combined fractional diagonal chromatography method that also allows identification of exact ubiquitination sites [234,235]. A direct approach known as UBAIT is based on the use of E3 enzyme-ubiquitin fusions [230]. This results in covalent binding of E3 to its ubiquitination target, which facilitates its purification. Derivatives of this technique are the TULIP (targets of ubiquitin ligases identified by proteomics) [236] and TULIP2 methodologies [237]. These techniques have been used successfully with the RING and HECT E3 enzymes. Their application to the study of NEL E3 should dramatically increase the number of candidate substrates for these enzymes. An associated task is the identification of the specific type of ubiquitin linkage for each target (monoubiquitination, K48 or K63 polyubiquitination, etc.) and its fate (proteasomal degradation or other). In addition to finding targets for the catalytic activity of NEL effectors, it is also interesting to identify interacting partners that are not ubiquitinated, since the physical interaction may be enough to alter the function or localization of these partners.

Structural information provided insight into the molecular mechanisms of autoinhibition and activation of NEL ligases from *Shigella* and *Salmonella*. However, this information is lacking for the *Ralstonia* and *Sinorhizobium* effectors. NopM from *S. fredii* has the domain composition found in typical members of the family, and hence the LRR domain is expected to play a role in inhibition of the NEL catalytic activity and in the interaction with substrates. More intriguing is the case of the four NEL effectors identified in *Ralstonia*, since they lack an LRR domain. New computational methods recently developed [238] should soon provide accurate predictions of the structures of these proteins that will help to understand their similarities and differences with typical members of the family. Furthermore, because E3 ligases of this family have sequences, structures, and mechanisms of action that are specific to Gram-negative bacteria, and since they play a relevant role in pathogenesis, they are attractive targets for the development of inhibitory drugs that would not have side effects in the host. These inhibitors would not limit the growth of pathogenic bacteria outside the host, minimizing the probability of selection of resistant variants.

The study of the degree of redundancy between effectors of this family that are expressed in the same bacteria is another topic of great interest. Functional specialization for each member of the family could be achieved through different patterns and timings of expression, specific localizations, or different abilities to interact with a certain host protein. Recent work highlights the drastic modification of the ubiquitination machinery at the cellular level in the context of bacterial infection [65]. However, several questions remain to be addressed. For instance, what is the impact of the modulation of this system at the organism level? How different are the ubiquitin hijacking strategies employed by pathogens in different cell types? Further work on the host–pathogen battle will provide insights not only on the molecular mechanisms of these fascinating bacterial molecules, but also on some aspects of host ubiquitin signaling.

## Figures and Tables

**Table 2:** Host targets of NEL T3SS effectors.

Organism	Effector Name	Main Characterized Binding Partners	
		No Ubiquitination Shown	Ubiquitination Shown
*S. enterica*	SlrP	ERdj3	Thioredoxin
	SspH1		PKN1
	SspH2	Profilins, filamin, SGT1	Nod1
*S. flexneri*	IpaH1.4	HOIL-1L	HOIP
	IpaH2.5	HOIL-1L	HOIP
	IpaH4.5	Rab31	p65, TBK1, RPN13, NLRP3
	IpaH7.8		Glomulin, GSDMB, GSDMD
	IpaH9.8		Ste7, U2AF^35^, NEMO, GBP1, GBP2, GBP4
	IpaHa		TRAF2

## Data Availability

Not applicable.
